# H_2_S Is a Potential Universal Reducing Agent for Prx6‐Type Peroxiredoxins

**DOI:** 10.1002/advs.202507214

**Published:** 2025-10-01

**Authors:** Lukas Lang, Laura Leiskau, Lea Bambach, Marcel Deponte

**Affiliations:** ^1^ Faculty of Chemistry Comparative Biochemistry RPTU Kaiserslautern D‐67663 Kaiserslautern Germany

**Keywords:** enzyme catalysis, hydrogen peroxide, hydrogen sulfide, kinetics, peroxiredoxin

## Abstract

The absence of a universal reducing agent distinguishes the Prx6‐type subfamily of peroxiredoxins from the structurally similar Prx1‐type subfamily. A likely explanation for the lack of reactivity of Prx6‐type enzymes with common reducing agents is that a histidyl residue at the bottom of the active‐site pocket traps the oxidized enzyme in an inaccessible fully‐folded protein conformation. Here, we analyzed the reduction of oxidized PfPrx6 from *Plasmodium falciparum* and human PrxVI by the hydrosulfide ion, HS^−^, as the smallest possible sulfur‐containing universal electron donor. We show that HS^−^ rapidly reacts with oxidized wild‐type PfPrx6 or human PrxVI (but not the histidyl mutants PfPrx6^H39Y^ or hPrxVI^H39Y^) with a second‐order rate constant of > 10^8^ m ^‒1^s^‒1^ at pH 7.4. The obtained protein‐hydropersulfide species is neither reduced by thioredoxin nor glutaredoxin and glutathione, but further reacts with an excess of HS^−^ with a second‐order rate constant around 10^4^ m ^‒1^s^‒1^, yielding the reduced enzyme. In summary, we identified HS^−^ as a highly reactive, potential universal electron donor for Prx6‐type enzymes. This study marks the starting point for the characterization of the complex reduction pathway of Prx6‐type enzymes with implications for H_2_S detoxification and redox signaling as well as iron‐sulfur and persulfide metabolism.

## Graphical abstract

We report the identification of H_2_S as reducing agent for Prx6‐type peroxiredoxins. In contrast to common physiological reducing agents of other peroxiredoxins, HS^−^ is small enough to access the active site of oxidized Prx6‐type enzymes in the fully‐folded protein conformation. Prx6‐type enzymes could therefore have novel functions in H_2_S detoxification, redox signaling, and/or iron‐sulfur and persulfide metabolism.

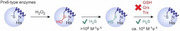

## 1 Introduction

The identification of a universal physiological reducing agent for oxidized Prx6‐type peroxiredoxins is an ongoing quest since the reported characterization of recombinant human PrxVI (hPrxVI) in 1998.^[^ ^1^ ^]^ Peroxiredoxins (EC 1.11.1.15) are highly abundant peroxidases in eukaryotes and prokaryotes that play a central role in peroxide detoxification and redox signaling.^[^ ^2^, ^3^, ^4^, ^5^, ^6^ ^]^ Furthermore, some peroxiredoxins can form decamers and higher oligomeric structures with chaperone activity.^[^ ^7^, ^8^, ^9^ ^]^ The catalytic cycle of peroxiredoxins requires a peroxidatic cysteinyl residue, which is buried in an active‐site pocket and reduces the peroxide substrate. The oxidized enzyme then usually undergoes a conformational change (local protein unfolding) so that its sulfenic acid becomes accessible to a resolving cysteinyl residue and/or common reducing agents such as thioredoxins (Trx), glutaredoxins (Grx), or glutathione (GSH).^[^ ^2^, ^3^, ^5^, ^10^ ^]^ Analogous to other peroxiredoxin subfamilies, Prx6‐type enzymes are peroxidases that reduce various hydroperoxides and peroxynitrous acid, yielding the enzyme sulfenic acid and either water, the corresponding alcohol, or nitrite.^[^ ^11^, ^12^, ^13^, ^14^, ^15^, ^16^, ^17^ ^]^ However, despite high structural similarity to Prx1‐type enzymes,^[^ ^10^ ^]^ Prx6‐type enzymes have a subfamily‐specific active‐site histidyl residue,^[^ ^18^, ^19^, ^20^ ^]^ and many oxidized Prx6‐type enzymes cannot be reduced by Trx, Grx, or GSH.^[^ ^1^, ^11^, ^12^, ^13^, ^14^, ^15^, ^17^, ^18^, ^20^, ^21^ ^]^ It is therefore unclear, how oxidized Prx6‐type enzymes are reduced again to complete the catalytic cycle. A few exceptions include hPrxVI, which is reduced by GSH in the presence of glutathione transferase P1‐1,^[^ ^22^ ^]^ and its yeast homologue, which is directly or indirectly connected to the mitochondrial glutathione pool.^[^ ^23^ ^]^ Successful reductions of oxidized Prx6‐type enzymes were usually detected for non‐physiological dithiothreitol (DTT) in contrast to other low‐molecular‐weight thiols.^[^ ^1^, ^11^, ^13^, ^14^, ^15^, ^16^, ^17^, ^18^, ^20^ ^]^ Ascorbate was also shown to reduce some oxidized Prx6 homologues in vitro,^[^ ^24^, ^25^ ^]^ and H_2_Se derived from selenocysteine was recently reported to form a thioperselenide with hPrxVI.^[^ ^26^, ^27^ ^]^ However, Prx6‐type enzymes are found in eukaryotes, bacteria, and archaea,^[^ ^10^, ^19^ ^]^ many of which have no corresponding glutathione transferase, or ascorbate or selenocysteine metabolism.^[^ ^5^, ^28^, ^29^ ^]^ Accordingly, several Prx6‐type enzymes, including PfPrx6 from the human malaria parasite *Plasmodium falciparum*, showed no activity with a glutathione transferase or ascorbate.^[^ ^1^, ^13^, ^15^, ^17^, ^20^, ^21^ ^]^ Here, we analyzed the reaction kinetics between oxidized PfPrx6 or hPrxVI and the H_2_S‐derived hydrosulfide ion, HS^−^, as a potential universal electron donor for oxidized Prx6‐type enzymes with implications for H_2_S detoxification and redox signaling as well as iron‐sulfur and persulfide metabolism.

## 2 Results and Discussion

### 2.1 Oxidized PfPrx6 and hPrxVI React Rapidly with HS^−^

**Figure 1:**
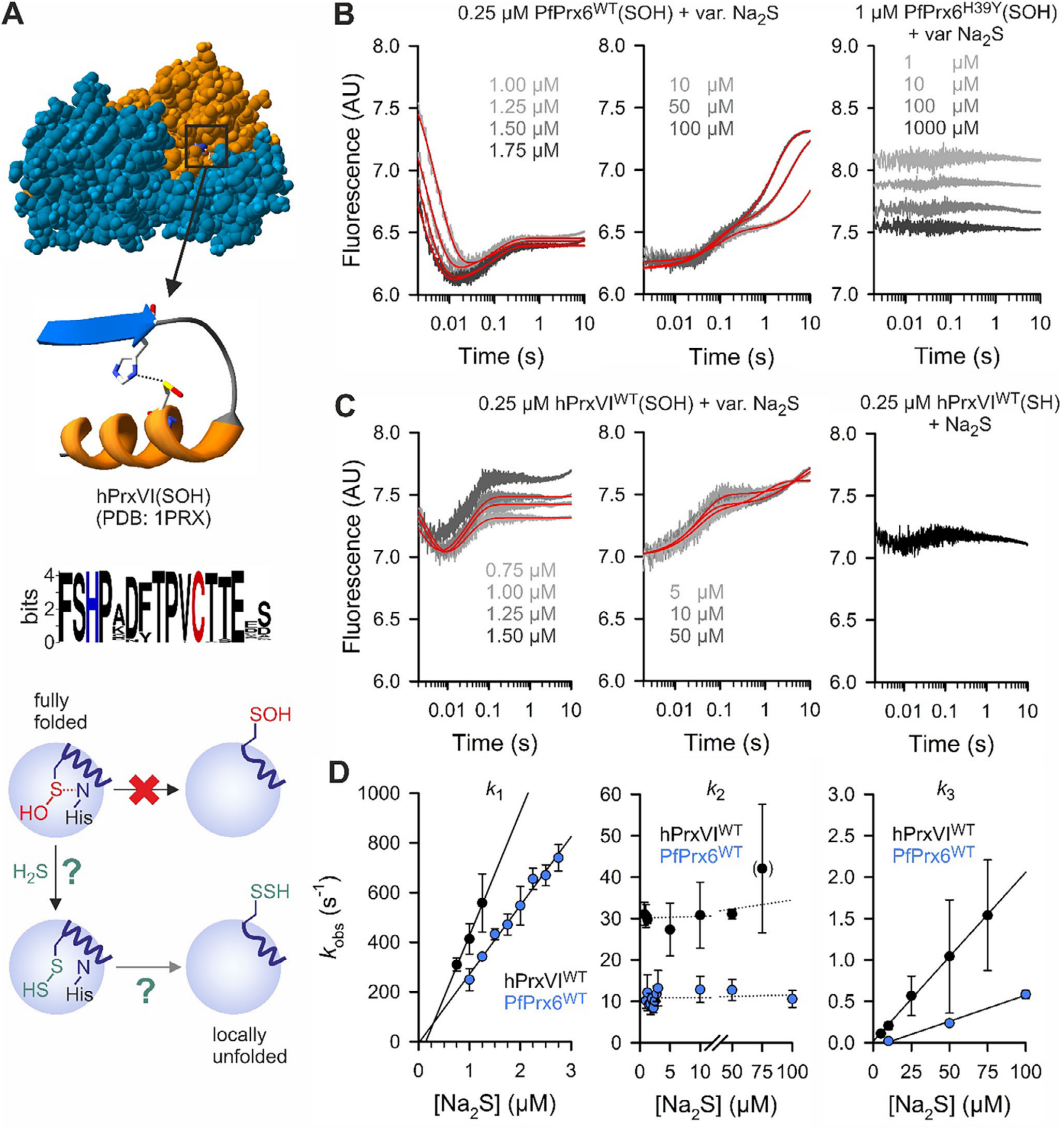
Rapid reduction of oxidized PfPrx6 and hPrxVI by HS^−^. A) Structure of oxidized homodimeric hPrxVI (top, PDB entry 1PRX), signature conservation graph for 5200 Prx6‐type enzymes (middle),^[^ ^19^ ^]^ and schematic summary of a potential HS^−^‐dependent reduction mechanism of Prx6‐type enzymes (bottom). The active‐site cysteinyl and histidyl residues are highlighted for which the electron densities of crystallized hPrxVI and ApPrx6 were interpreted as a hydrogen bond or a hypervalent sulfur species, respectively.^[^ ^30^, ^32^ ^]^ B) Representative kinetic traces of stopped‐flow measurements with oxidized PfPrx6^WT^ (left panels) and PfPrx6^H39Y^ (right panel) at pH 7.4 and 25 °C. Double exponential fits are shown in red. C) Representative kinetic traces of stopped‐flow measurements with oxidized (left panels) and reduced hPrxVI^WT^ (right panel) at pH 7.4 and 25 °C. Double exponential fits are shown in red. All kinetic traces in panels B and C were averaged from technical triplicate measurements. D) Secondary plots of the *k*_obs_ values with S.D. from the fits in panels B and C determined from three independent biological replicates including at least three technical replicates each (sample size *n* ≥ 3 × 3). Mean rate constants with S.D. were determined from the slopes or the y‐axis intercepts of the linear fits from three independent biological replicates and are shown in Table 1.

We previously showed that oxidized PfPrx6 most likely stays in a trapped, fully‐folded enzyme conformation involving an interaction between the sulfenic‐acid form of the peroxidatic cysteinyl residue and the Prx6‐specific histidyl residue at the bottom of the active‐site pocket.^[^ ^12^, ^18^, ^20^ ^]^ These findings were in accordance with crystal structures of oxidized hPrxVI and ApPrx6 from the archaeon *Aeropyrum pernix*, revealing either a hydrogen bond or a hypervalent sulfur species that involves the active‐site cysteinyl and histidyl residue (**Figure** **1**A).^[^ ^30^, ^31^, ^32^ ^]^ Since oxidized PfPrx6 could only be reduced by DTT but not by other small thiols or ascorbate,^[^ ^20^ ^]^ we decided to test HS^−^ as the smallest possible sulfur‐containing universal electron donor for inaccessible, fully‐folded Prx6‐type enzymes using stopped‐flow kinetic measurements (Figure 1A,B). Since the p*K*_a_ values of H_2_S are 7.0 and >14,^[^ ^33^ ^]^ freshly dissolved Na_2_S can be used as an HS^−^ source. Mixing oxidized wild‐type PfPrx6 (PfPrx6^WT^) in the first syringe with variable concentrations of Na_2_S in the second syringe resulted in up to three distinct reaction phases (Figure 1B). Similar results were obtained for recombinant wild‐type hPrxVI (hPrxVI^WT^), whereas no activity was detected for the active‐site mutant hPrxVI^C47S^, as shown in Figure 1C and Figure S1A (Supporting Information), respectively. An extremely rapid Na_2_S concentration‐dependent decrease of tryptophan fluorescence was observed within 20–30 ms (Figure 1B), resulting in a second‐order rate constant of (2.8 ± 0.2) × 10^8^ m ^‒1^s^‒1^ for PfPrx6^WT^ at pH 7.4 (Figure 1D and **Table** **1**), which corresponds to a pH‐independent value of 3.9 × 10^8^ m ^‒1^s^‒1^ (based on the HS^−^ protonation state). For hPrxVI^WT^, the first phase occurred during the dead‐time for Na_2_S concentrations ≥ 10 µm and was even faster with pH‐dependent and ‐independent second‐order rate constants of (5.0 ± 1.2) × 10^8^ and 7.0 × 10^8^ m ^‒1^s^‒1^, respectively (Figure 1C,D and Table 1). Decreasing the pH from 7.4 to 6.4 also decreased the rate constant of the first phase for hPrxVI^WT^ to (1.5 ± 0.3) × 10^8^ m ^‒1^s^‒1^, whereas the pH‐independent value of 7.5 × 10^8^ m ^‒1^s^‒1^ remained similar (Table 1; Figure S1B,E, Supporting Information). A subsequent increase of fluorescence during a second reaction phase did not depend on the Na_2_S concentration, yielding a rate constant around 11 s^‒1^ for PfPrx6^WT^ or 30 s^‒1^ for hPrxVI^WT^. A third phase was observed at higher Na_2_S concentrations, resulting in an increase of tryptophan fluorescence with a second‐order rate constant around (6.3 ± 0.3) × 10^3^ m ^‒1^s^‒1^ for PfPrx6^WT^ or (2.0 ± 0.4) × 10^4^ m ^‒1^s^‒1^ for hPrxVI^WT^ (Figure 1B–D and Table 1). Measurements with Li_2_S or thioacetamide (TAA) as alternative HS^−^ sources resulted in similar rate constants (Table 1; Figure S2, Supporting Information). The kinetic phases for PfPrx6^WT^ and hPrxVI^WT^ depended on the presence of the conserved histidyl residue and were lost for oxidized PfPrx6^H39Y^ and hPrxVI^H39Y^ as shown in Figure 1B and Figure S1C (Supporting Information), respectively. The results are consistent with the much slower DTT‐dependent (*k* = 322 m ^‒1^s^‒1^) reduction of oxidized PfPrx6, which also resulted in a decreased tryptophan fluorescence and required the presence of the histidyl residue.^[^ ^20^ ^]^ In contrast, we previously showed by circular dichroism spectroscopy and stopped‐flow kinetic measurements that PfPrx6^H39Y^ is correctly folded and that the formation of the sulfenic acids of PfPrx6^WT^ and PfPrx6^H39Y^ results in similar changes of tryptophan fluorescence with almost identical rate constants.^[^ ^12^, ^20^ ^]^ Thus, the negative result for the HS^−^‐dependent reduction of oxidized PfPrx6^H39Y^ in Figure 1B was not caused by altered tryptophan fluorescence properties and the reduction (but not the oxidation) of PfPrx6 requires the conserved histidyl residue. Furthermore, a negative control with reduced hPrxVI^WT^ and Na_2_S revealed no reaction and therefore contradicts the presence of other (contaminating) reactive sulfur species that might have oxidized the protein or altered the fluorescence in general (Figure 1C, right panel). In summary, oxidized PfPrx6^WT^ and hPrxVI^WT^, but not PfPrx6^H39Y^ and hPrxVI^H39Y^, react rapidly with HS^−^ as a potential universal electron donor for Prx6‐type enzymes.

**Table 1:** Rate constants *k*_1_, *k*_2_, and *k*_3_ for the first, second, and third reaction phase from Figure 1 and Figures S1 and S2 (Supporting Information) for the reactions of oxidized PfPrx6^WT^, hPrxVI^WT^, or hPrxVI^C91S^ with alternative HS^−^ sources at pH 7.4 or 6.4 and 25 °C.

			Rate constant		
**Enzyme**	**Substrate**	**pH**	*k*_1_ (M^−1^s^−1^)	*k*_2_ (s^−1^)	*k*_3_ (M^−1^s^−1^)
PfPrx6^WT^	Na_2_S	7.4	(2.8 ± 0.2) × 10^8^	≈ 11	(6.3 ± 0.3) × 10^3^
	Li_2_S		(2.0 ± 0.1) × 10^8^	≈ 11	(4.8 ± 0.5) × 10^3^
	TAA^a)^		(4.1 ± 1.1) × 10^7^	≈ 19	(1.9 ± 0.3) × 10^3^
hPrxVI^WT^	Na_2_S	7.4	(5.0 ± 1.2) × 10^8^	≈ 30	(2.0 ± 0.4) × 10^4^
		6.4	(1.5 ± 0.3) × 10^8^	≈ 33	(3.1 ± 0.5) × 10^3^
hPrxVI^C91S^	Na_2_S	7.4	(3.3 ± 0.1) × 10^8^	≈ 30	(1.5 ± 0.1) × 10^4^

^a)^ The in situ formation of HS^−^ by a one‐hour acid treatment of TAA might have been incomplete and/or resulted in additional sulfur species.

### 2.2 Working Model for the HS^−^‐Dependent Reduction of PfPrx6 and hPrxVI

**Figure 2:**
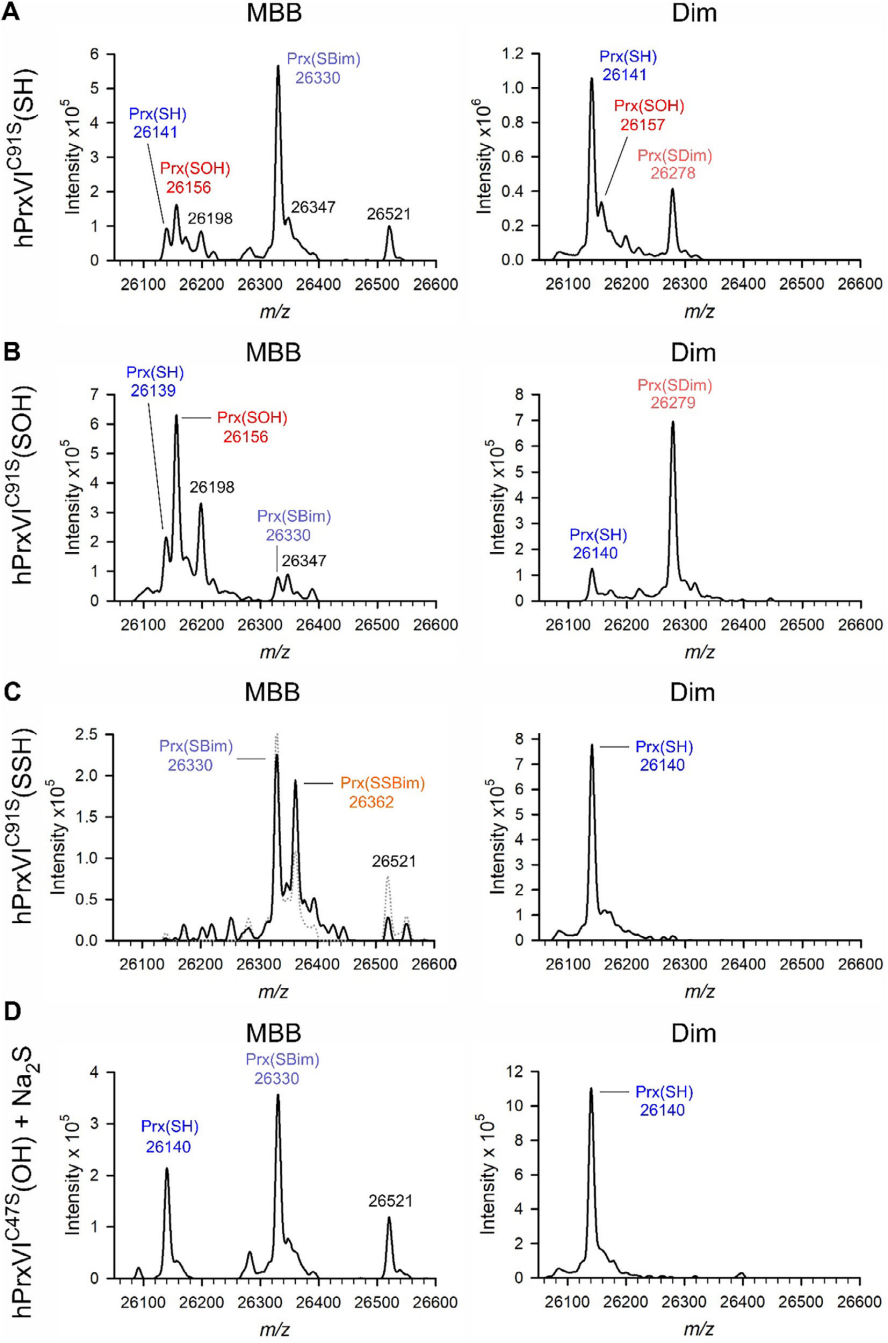
Mass spectrometric detection of the reduced, oxidized, and hydropersulfide species of hPrxVI^C91S^. A) Whole protein mass spectra of the DTT‐reduced enzyme (without pretreatment with H_2_O_2_) that was incubated with either MBB (left) or Dim (right). B) Whole protein mass spectra of the H_2_O_2_‐oxidized enzyme species of hPrxVI^C91S^ that was incubated with either MBB (left) or Dim (right). C) Whole protein mass spectra of the hydropersulfide species of hPrxVI^C91S^ that was incubated with either MBB (left) or Dim (right). D) Inactive hPrxVI^C47S^ was treated as in panel C and served as a negative control. Calculated masses of the reduced, oxidized, and hydropersulfide species of hPrxVI^C91S^ or hPrxVI^C47S^ are 26 141, 26 157, and 26 173 Da, respectively. MBB was used for the alkylation of the thiol and hydropersulfide species, yielding the alkylated species Prx(SBim) and Prx(SSBim) with a calculated mass shift of +190 Da. Dim was used for the detection of the H_2_O_2_‐oxidized species, yielding the alkylated species Prx(SDim) with a calculated mass shift of +122 Da. Please note that protein(SSBim) species can be partially converted to protein(SBim) species by a tautomerization/desulfurization mechanism.^[^ ^34^ ^]^ Sample size for each measurement *n* = 1.

To interpret the observed reactions from Figure 1B,C, we performed a mass spectrometric analysis of the different redox species of a single‐cysteine mutant of hPrxVI (hPrxVI^C91S^) (**Figure** **2**). In contrast to PfPrx6^WT^, which has eight cysteinyl residues that complicate data analyses, hPrxVI^C91S^ has no other cysteinyl residue than the one in the active site and showed very similar changes of tryptophan fluorescence and kinetics in stopped‐flow measurements with Na_2_S (Table 1; Figure S1D,E, Supporting Information). We compared the DTT‐reduced enzyme species (Figure 2A), the oxidized enzyme species that was generated with an equimolar amount of H_2_O_2_ (Figure 2B), and the potential reaction intermediate of the HS^−^‐reduced enzyme species that was generated by successive oxidation with one equivalent H_2_O_2_ and reduction with one equivalent Na_2_S (Figure 2C). Active‐site mutant hPrxVI^C47S^ was incubated as hPrxVI^C91S^ in Figure 2C and served as a negative control (Figure 2D). The protein samples were either incubated with monobromobimane (MBB), which alkylates thiols and hydropersulfides,^[^ ^34^ ^]^ or dimedone (Dim), which can be used for the alkylation of sulfenic acids or sulfenamides.^[^ ^35^, ^36^ ^]^ As expected, DTT‐reduced hPrxVI^C91S^ was predominantly alkylated by MBB whereas oxidized hPrxVI^C91S^ was predominantly alkylated by Dim (Figure 2A,B). No alkylation by Dim was detected for the oxidized enzyme species that was reduced with one equivalent of Na_2_S (Figure 2C, right panel). In contrast, a novel enzyme species with an additional sulfur atom was alkylated by MBB in accordance with the formation of the hydropersulfide species hPrxVI^C91S^(SSH) (Figure 2C, left panel, solid line). Increasing the Na_2_S concentration to ten equivalents increased the intensity of the labeled reduced enzyme species and decreased the intensity of the labeled hydropersulfide species (Figure 2C, left panel, dotted line), whereas no hydropersulfide species was detected for hPrxVI^C47S^ (Figure 2D). The hydropersulfide species of hPrxVI^C91S^ resembles the recently detected thioperselenide species hPrxVI^C91S^(SSeH) that was formed with H_2_Se.^[^ ^26^, ^27^ ^]^

While the exact intermediates of the complex reduction pathway involving the active‐site cysteinyl and histidyl residue remain to be characterized in more detail, our whole protein mass spectra support the following interpretation of the fluorescence changes for the HS^−^‐dependent reduction of PfPrx6 and hPrxVI in Figure 1B,C: For the first reaction phase, we suggest a direct reaction between HS^−^ and the potentially hypervalent sulfur atom of the trapped oxidized species of both Prx6‐type enzymes (Figure 1A). Alternatively, following the elimination of water, HS^−^ could attack the sulfenamide between the active‐site cysteinyl and histidyl residue. In both alternatives, the protein hydropersulfides PfPrx6^WT^(SSH) and hPrxVI^WT^(SSH) are the resulting enzyme species of the first reaction phase. Please note that analogous to the trapped, fully‐folded sulfenic acid species of PfPrx6^WT^, which reacts with HS^−^ (Figure 1B) but not cysteine,^[^ ^20^ ^]^ hPrxVI was shown to react with H_2_Se but not selenocysteine (which was explained by a steric inaccessibility of the sulfenic acid species).^[^ ^26^ ^]^ Furthermore, similar to the absent reaction of oxidized PfPrx6^H39Y^ and hPrxVI^H39Y^ with HS^−^ (Figure 1B; Figure S1C, Supporting Information), the presence of the active‐site histidyl residue was a prerequisite for the selenium transfer between hPrxVI and glutathione peroxidase 4 in SK‐N‐DZ cells.^[^ ^26^ ^]^ It remains to be studied whether the essential histidyl residue i) activates the electrophilic sulfur atom of the oxidized enzyme, ii) positions the nucleophile and electrophile, and/or iii) acts as an acid‐base catalyst during the first reaction phase. In contrast to PfPrx6 and hPrxVI, the formation of a hydropersulfide between the oxidized 1‐Cys peroxiredoxin MtAhpE from *Mycobacterium tuberculosis* and Na_2_S was much slower with a second‐order rate constant of (1.4 ± 0.3) × 10^3^ m ^‒1^s^‒1^.^[^ ^37^ ^]^ A plausible explanation for the lower reactivity could be that a tyrosyl or phenylalanyl residue replaces the active‐site histidyl residue in AhpE‐type enzymes.^[^ ^19^ ^]^ The second reaction phase in Figure 1B,C did not depend on the HS^−^ concentration and might indicate an intramolecular rearrangement reflecting an equilibrium between a potential reaction product with a hypervalent sulfur atom and the free hydropersulfide species of PfPrx6^WT^(SSH) or hPrxVI^WT^(SSH) in the fully‐folded protein conformation. The third phase could reflect the slower formation of fully reduced PfPrx6^WT^ or hPrxVI^WT^ by excess Na_2_S, yielding hydrogen disulfide (H_2_S_2_). The final fluorescence after reaction with an excess of Na_2_S in Figure 1B was slightly below the initial fluorescence of the oxidized enzyme, which is in accordance with a decreased fluorescence of the reduced enzyme as reported previously.^[^ ^12^ ^]^ In summary, our kinetic and mass spectrometry data support a coherent working model for the complex reduction pathway of Prx6‐type enzymes.

### 2.3 Excess HS^−^ Fully Reduces Oxidized PfPrx6

**Figure 3:**
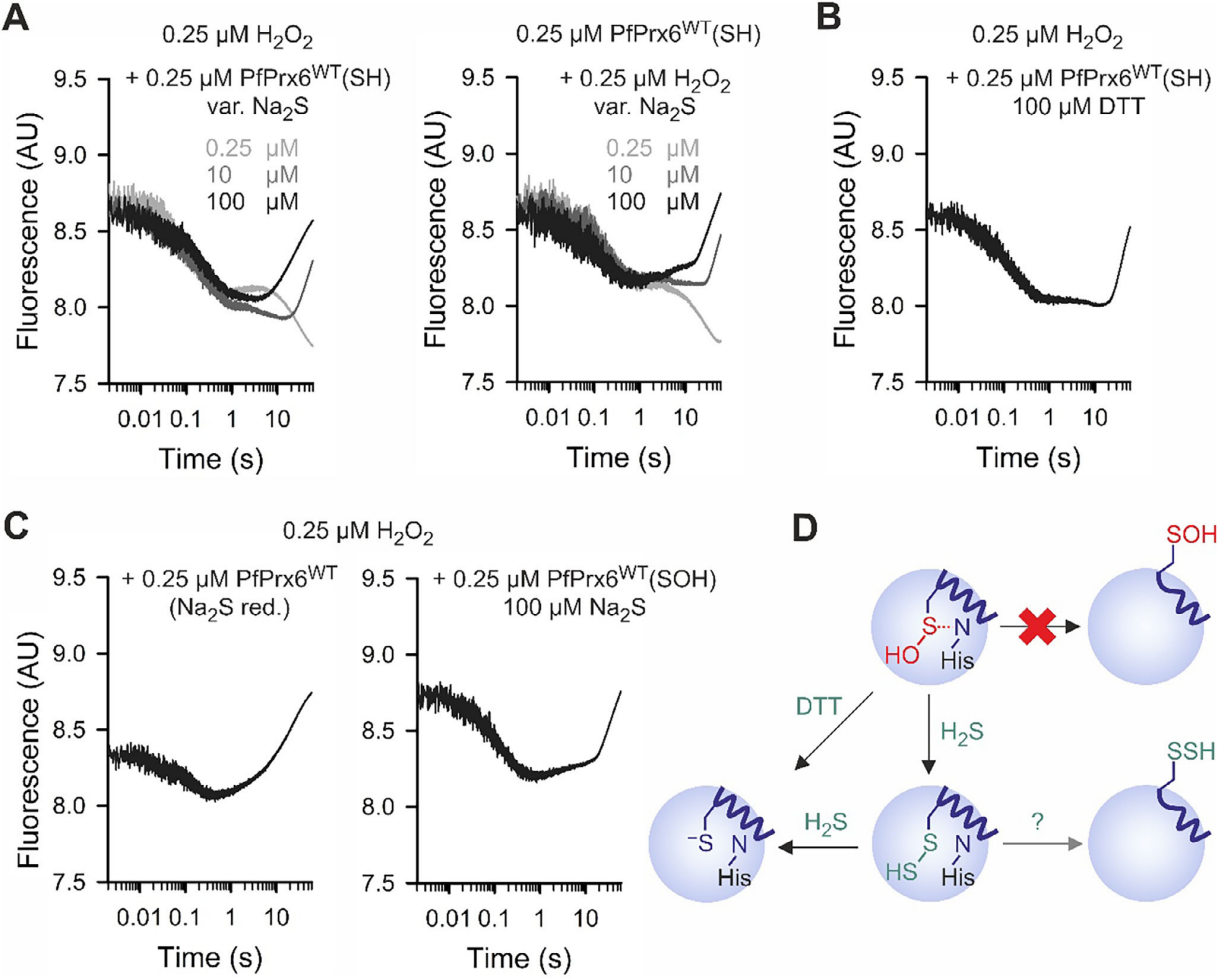
Excess HS^−^ fully reduces PfPrx6. A) Representative kinetic traces of single turnover stopped‐flow measurements with either one equivalent of H_2_O_2_ in the first syringe and reduced PfPrx6^WT^ and the indicated concentrations of Na_2_S in the second syringe (left panel) or one equivalent of reduced PfPrx6^WT^ in the first syringe and freshly mixed H_2_O_2_ and the indicated concentrations of Na_2_S in the second syringe (right panel). B) Control experiment as in panel A with DTT replacing Na_2_S. C) Representative kinetic traces for the reaction between one equivalent H_2_O_2_ in the first syringe and PfPrx6^WT^ that was oxidized and subsequently reduced with an excess of Na_2_S in the second syringe. Excess Na_2_S was either removed on a PD‐10 column (left panel) or was still present in the reaction mixture (right panel). D) Schematic summary of the results. All concentrations indicate final concentrations in the mixing chamber. All experiments were performed at pH 7.4 and 25 °C. All kinetic traces in panels A–C were averaged from technical triplicate measurements and all results were confirmed in independent duplicate biological replicates with three technical replicates each (sample size *n* = 2 × 3).

To test our working model and to analyze if fully reduced PfPrx6^WT^ is generated in the presence of Na_2_S, we performed single‐turnover experiments and additional controls (**Figure** **3**). For the single‐turnover experiments, we either mixed H_2_O_2_ in the first syringe and reduced PfPrx6^WT^ with Na_2_S in the second syringe (Figure 3A, left) or reduced PfPrx6^WT^ in the first syringe and freshly mixed H_2_O_2_ and Na_2_S in the second syringe (Figure 3A, right). The alternative experimental designs were chosen to exclude a reaction between reduced PfPrx6^WT^ and potential polysulfides that might be present as impurities or that can be formed when H_2_O_2_ and Na_2_S are incubated for more than a few seconds.^[^ ^33^ ^]^ We observed similar changes of tryptophan fluorescence for both experimental designs, starting with a rapid loss of fluorescence (Figure 3A), which probably reflects the formation of the sulfenic acid species of PfPrx6^WT^ as shown previously.^[^ ^12^ ^]^ While a further loss of fluorescence was detected after seconds with an equimolar concentration of Na_2_S in accordance with the loss of fluorescence in Figure 1B, a concentration‐dependent increase of fluorescence was observed with an excess of Na_2_S (Figure 3A). We interpret the two alternatives of the final phase in Figure 3A as the formation of the hydropersulfide species with one equivalent of HS^−^ (which presumably requires a rate‐limiting conformational change or rearrangement after formation of the sulfenic acid species of PfPrx6^WT^),^[^ ^12^, ^20^ ^]^ and the additional formation of the fully reduced enzyme by another equivalent of HS^−^ at higher Na_2_S concentrations. Similar changes in tryptophan fluorescence were obtained with an excess of DTT instead of an excess of Na_2_S (Figure 3B), yielding the initial fluorescence at the end of a single‐turnover experiment in accordance with the fully reduced enzyme. To show that the resulting enzyme species is indeed fully reduced PfPrx6^WT^, we analyzed its reactivity with H_2_O_2_ as a control (Figure 3C). Pre‐incubation of oxidized PfPrx6^WT^ with an excess of Na_2_S resulted in an enzyme species with identical kinetics with H_2_O_2_ as in ref.[12] when excess Na_2_S was removed (Figure 3C, left). Here, the fluorescence increased beyond the initial fluorescence probably because of a conformational change or intramolecular rearrangement of the oxidized enzyme.^[^ ^12^ ^]^ In contrast, pre‐incubation of oxidized PfPrx6^WT^ with an excess of Na_2_S resulted in an enzyme species with identical kinetics with H_2_O_2_ as in Figure 3A when excess Na_2_S was present (Figure 3C, right). Here, the initial and final fluorescence were identical in accordance with a reduced enzyme species before and after the reaction. In summary, while the exact intermediates of the complete reduction pathway remain to be characterized in more detail, our single‐turnover experiments and controls support our working model and confirmed that the hydropersulfide species PfPrx6^WT^(SSH) can be reduced by an excess of HS^−^, yielding the fully reduced enzyme (Figure 3D).

### 2.4 PfPrx6 hydropersulfide Does not React with the Glutathione or Thioredoxin System

**Figure 4:**
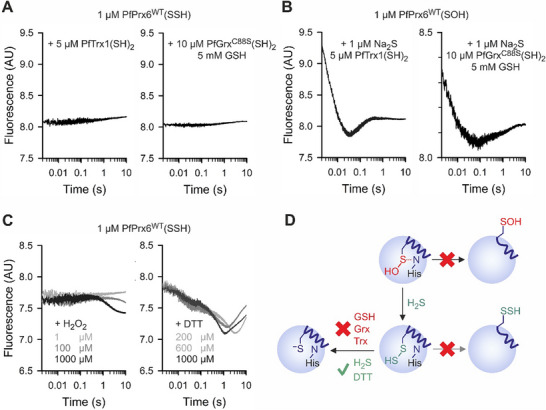
PfPrx6^WT^(SSH) is not reduced by the thioredoxin or glutathione system. Representative kinetic traces of stopped‐flow measurements with A) PfPrx6^WT^(SSH) or B) PfPrx6^WT^(SOH) and the indicated reductants. C) Representative kinetic traces for the reaction between PfPrx6^WT^(SSH) and H_2_O_2_ or DTT. D) Schematic summary of the results. All experiments were performed at pH 7.4 and 25 °C. All kinetic traces in panels A–C were averaged from technical triplicate measurements and all results were confirmed in duplicate biological replicates with three technical replicates each (sample size *n* = 2 × 3).

Next, we tested whether the (free or hypervalent) hydropersulfide species of PfPrx6^WT^ reacts with alternative reducing agents including the glutathione and thioredoxin systems (**Figure** **4**).^[^ ^5^, ^21^ ^]^ We therefore incubated oxidized PfPrx6^WT^ with one equivalent of Na_2_S and subsequently mixed the resulting enzyme species with either reduced thioredoxin (PfTrx1) or glutaredoxin (PfGrx) and GSH (Figure 4A). Mutant PfGrx^C88S^ was used because it can react via a mono‐ or dithiol mechanism with glutathione disulfides and protein disulfides without potential side reactions involving a third cysteinyl residue.^[^ ^38^, ^39^, ^40^ ^]^ Since no change in fluorescence was detected, we excluded potential rapid reactions during the dead‐time of the instrument by mixing oxidized PfPrx6^WT^ in the first syringe and Na_2_S and either reduced PfTrx1 or PfGrx^C88S^/GSH in the second syringe (Figure 4B). No additional phases were observed as compared to Figure 1B, indicating no reaction between PfPrx6^WT^(SSH) and PfTrx1 or PfGrx^C88S^/GSH. This is in contrast to the efficient reduction of several protein hydropersulfides including PTP1B(SSH) and HSA(SSH) by Grx/GSH or Trx.^[^ ^41^, ^42^, ^43^ ^]^ We also tested the reaction between PfPrx6^WT^(SSH) and H_2_O_2_ or DTT (Figure 4C). In contrast to reduced PfPrx6^WT^,^[^ ^12^ ^]^ no change in fluorescence occurred with H_2_O_2_ unless millimolar concentrations were used, resulting in a slow reaction (Figure 4C, left panel). Complex changes in fluorescence were observed for the reaction of PfPrx6^WT^(SSH) with DTT. In contrast to the DTT‐dependent reduction of PfPrx6^WT^(SOH),^[^ ^20^ ^]^ the reaction with PfPrx6^WT^(SSH) occurred much faster, and the number of phases varied depending on the DTT concentration used (Figure 4C, right panel). The reactivity of PfPrx6^WT^(SSH), or the lack thereof, and the observed amplitudes overall suggest that the enzyme rests in the fully‐folded conformation. Likewise, incubation of hPrxVI with selenite and GSH was shown to yield exclusively the thioperselenide species PrxVI(SSeH) but no detectable adduct with glutathione.^[^ ^27^ ^]^ In summary, the reactivity of PfPrx6^WT^(SSH) is very different from reduced PfPrx6^WT^. PfPrx6^WT^(SSH) can be reduced by Na_2_S or rapidly react in a complex mechanism with DTT, but neither accepts electrons from PfTrx1 or PfGrx^C88S^/GSH nor reacts with H_2_O_2_ as an oxidant (Figure 4D).

### 2.5 Physiological Implications of HS^−^ as a Reducing Agent for Prx6‐Type Enzymes

While hPrxVI was recently shown to react with selenocysteine‐derived H_2_Se with implications for ferroptosis,^[^ ^26^, ^27^ ^]^ our data on PfPrx6 and hPrxVI suggest that HS^−^ is a potential universal reducing agent for Prx6‐type peroxiredoxins. Unlike ascorbate, selenocysteine, or even GSH,^[^ ^5^, ^28^, ^29^ ^]^ H_2_S and its anions HS^−^ and S^2−^ are present in all eukaryotes, bacteria, and archaea because of various non‐enzymatic and enzymatic sources and the fundamental biochemistry of iron‐sulfur clusters.^[^ ^33^, ^44^, ^45^, ^46^, ^47^ ^]^ In addition to its role as a metabolite, H_2_S can serve as a signaling molecule or be an external (toxic) gas.^[^ ^44^, ^48^, ^49^ ^]^ Depending on the experimental setup and chosen model system, physiological H_2_S concentrations are most likely below 1 µm and closer to 10 nm, although intracellular gradients may exist and epithelia can be exposed to much higher extracellular concentrations.^[^ ^44^, ^48^, ^50^, ^51^ ^]^ A role of Prx6‐type enzymes for H_2_S detoxification had been previously suggested based on high enzyme levels in lung tissue and epithelia as well as semi‐quantitative in vitro data with bovine PrxVI.^[^ ^16^ ^]^ However, the 2‐Cys Prx6‐type enzyme from the annelid *Arenicola marina*, which lives in sediments that can contain high H_2_O_2_ and H_2_S concentrations, showed no activity in a steady‐state assay with both redox reagents.^[^ ^14^ ^]^ Such negative results do not exclude a rapid reaction of the oxidized enzyme with HS^−^ and might be explained by i) an enzyme inactivation due to hyperoxidation, ii) a relatively slow second reduction of the protein hydropersulfide by HS^−^, or iii) the conversion of the sulfenic acid form of the studied 2‐Cys Prx6‐type enzyme via its hydropersulfide species to its disulfide form without a net consumption of H_2_S. While we identified HS^−^ as a very efficient electron donor for the first reduction step of oxidized Prx6‐type enzymes with a second‐order rate constant > 10^8^ m ^‒1^s^‒1^, the second reduction by HS^−^ appears to be rather slow at physiological HS^−^ concentrations. For example, reaction rates for the first and second reduction of hPrxVI at 0.01–1.0 µm H_2_S are *v*_1_ = (5‒500) × [hPrxVI(OH)] (M/s) and *v*_2_ = (2‒200) × 10^−4^ × [hPrxVI(SSH)] (M/s), respectively. For comparison: *v*_1_ for the reduction of the sulfenic acid of the 1‐Cys Prx5‐type model enzyme PfAOP by 1‒10 mm GSH is (600‒6000) × [PfAOP(OH)] (M/s) and *v*_2_ for the reduction of the glutathionylated enzyme by 1 µM PfGrx is 0.1 × [PfAOP(SSG)] (M/s).^[^ ^20^, ^52^ ^]^ Trapping 1‐Cys Prx6‐type enzymes in the fully‐folded conformation could therefore be a prerequisite to prevent competing reactions with other reductants at physiological H_2_S concentrations, in contrast to promiscuous 1‐Cys Prx5‐type enzymes.^[^ ^20^ ^]^ Considering the slower second reaction, full reduction of Prx6‐type enzymes by H_2_S requires either high local H_2_S concentrations or reaction times of several minutes to hours (which might be a desirable property for signal transduction involving the hydropersulfide enzyme species). The potential product of the second reduction is H_2_S_2_, which might react in vivo with millimolar GSH and serve as a source for glutathione hydropersulfide (GSSH). GSSH could then react either with protein thiols, yielding protein hydropersulfides, or with another molecule of GSH, yielding H_2_S and GSSG. A Prx6‐ and HS^−^‐dependent reduction of H_2_O_2_ yielding H_2_S_2_ could therefore explain the net formation of GSSG detected in yeast mitochondria.^[^ ^23^ ^]^ In conclusion, Prx6‐type enzymes might i) buffer fluctuations in (toxic) H_2_S levels, ii) act as H_2_S sensors that also integrate H_2_O_2_ signals, iii) require another partner for local unfolding to facilitate the reduction of the hydropersulfide species (as shown for the sulfenic acid species of hPrxVI and glutathione transferase P1‐1),^[^ ^22^ ^]^ and/or iv) convert H_2_O_2_ and two molecules of H_2_S to water and H_2_S_2_ as a source of physiological persulfides. Prx6‐type enzymes are often cytosolic. A Prx6‐dependent cytosolic generation of physiological persulfides is therefore particularly intriguing considering their emerging roles in redox biochemistry and their therapeutic potential for (patho)biochemical processes.^[^ ^53^, ^54^, ^55^, ^56^ ^]^ Follow‐up studies are necessary to characterize the exact reaction mechanism for the HS^−^‐dependent reduction of PfPrx6, hPrxVI, and other Prx6‐type enzymes, and to elucidate the physiological relevance of Prx6‐type enzymes for the detoxification of external or internal H_2_S, H_2_S‐dependent redox signaling, as well as iron‐sulfur and persulfide metabolism.

## 3 Experimental Section

### Materials

99.98% Lithium sulfide (Li_2_S), 98% sodium sulfide (Na_2_S), dithiothreitol (DTT), reduced l‐glutathione (GSH), and 99% thioacetamide (TAA) were from Sigma–Aldrich, diethylenetriaminepentaacetic acid (DTPA) was from Carl Roth, H_2_O_2_ from VWR, and isopropyl‐*β*‐d‐1‐thiogalactopyranoside (IPTG) was from Serva. Dimedone (Dim) was from Tokyo Chemical Industry and monobromobimane (MBB) was from Cayman Chemical. Restriction enzymes and DNA polymerase were from New England Biolabs (NEB).

### Molecular Biology, Heterologous Expression, and Protein Purification

The gene encoding hPrxVI was PCR‐amplified with Phusion HF DNA polymerase using forward primer GATC *CCATGG* GACCCGGAGGTCTGCTTCTCG, reverse primer GATC *CTCGAG* AGGCTGGGGTGTGTAGCGG, and plasmid p413/*HSPRDX6‐FLAG* as a template.^[^ ^23^ ^]^ The product (which encodes an additional prolyl residue at the N‐terminus due to the *Nco*I restriction site) was subcloned into the *Nco*I and *Xho*I restriction sites of pET28a/*PFPRX6‐His6* in *Escherichia coli* strain XL1‐Blue, yielding pET28a/*HSPRXVI‐His6*. Mutants pET28a/*HSPRXVI^C91S^‐His6*, pET28a/*HSPRXVI^C47S^‐His6*, and pET28a/*HSPRXVI^H39Y^‐His6* were generated by site‐directed mutagenesis using forward primers C91S_fwd_ (CTTACAAT*AGC*GAAGAGCCCAC), C47S_fwd_ (CTTTACCCCAGTGT*C*CACCACAGAGCTTG), and H39Y_fwd_ (GGCATTCTCTTCTCC*T*ACCCTCGGGACTTTAC) and reverse primers C91S_rev_ (GTGGGCTCTTC*GCT*ATTGTAAG), C47S_rev_ (CAAGCTCTGTGGTG*G*ACACTGGGGTAAAG), and H39Y_rev_ (GTAAAGTCCCGAGGGT*A*GGAGAAGAGAATGCC). Non‐mutated template plasmid was digested with *Dpn*I before transformation of strain XL1‐Blue. The correct sequences were confirmed by Sanger sequencing of both strands (Microsynth Seqlab). Recombinant plasmid pET28‐encoded C‐terminally LEH_6_‐tagged PfPrx6^WT^, PfPrx6^H39Y^, hPrxVI^WT^, hPrxVI^C47S^, hPrxVI^H39Y^, and hPrxVI^C91S^ were produced in *E. coli* strain SHuffle T7 express (NEB) at 30 °C.^[^ ^12^, ^20^ ^]^ Plasmid pQE30‐encoded N‐terminally MRGSH_6_GS‐tagged PfGrx^C88S^ and PfTrx1 were produced in *E. coli* strain XL1‐Blue at 37 °C as previously described.^[^ ^20^, ^38^, ^57^ ^]^ Gene expression was induced at an OD_600nm_ of 0.5 with 0.5 mm IPTG for 4 h. *E. coli* cultures were then cooled in an ice‐water bath for 15 min and centrifuged (15 min, 4 °C, 3750×g). Cell pellets were resuspended in ice‐cold buffer I (20 mm imidazole, 100 mm Na_x_H_y_PO_4_, 300 mm NaCl, pH 8.0 at 4 °C). Cell suspensions were stirred on ice with DNaseI and 10 mg lysozyme for 45 min and then sonicated. The cell lysates were centrifuged (30 min, 4 °C, 10 000×g) and the supernatants loaded on pre‐equilibrated Ni‐NTA agarose (Qiagen) columns. The columns were washed with 15 column volumes of ice‐cold buffer I and proteins were eluted with ice‐cold buffer II (200 mm imidazole, 100 mm Na_x_H_y_PO_4_, 300 mm NaCl, pH 8.0 at 4 °C). The purity of the eluates was confirmed by analytical SDS‐PAGE (Figure S3, Supporting Information). Activities of PfPrx6^WT^, PfPrx6^H39Y^, hPrxVI^WT^, hPrxVI^H39Y^, and hPrxVI^C91S^ were confirmed with H_2_O_2_, the activity of PfTrx1 was confirmed with 5,5′‐dithiobis‐(2‐nitrobenzoic acid), and the activity of PfGrx^C88S^ was confirmed with bis(2‐hydroxyethyl)disulfide as described previously.^[^ ^12^, ^20^, ^58^ ^]^

### Sample Preparation

Freshly purified enzymes were reduced with 5 mm DTT for 30 min on ice. Excess DTT and imidazole were removed on a PD‐10 desalting column (Merck) and reduced proteins were eluted with 3.5 mL ice‐cold assay buffer (100 mm Na_x_H_y_PO_4_, 0.1 mm DTPA, pH 7.4 at 25 °C). Protein concentrations were determined spectrophotometrically at 280 nm using the corresponding extinction coefficients of 30 940 m ^‒1^cm^‒1^ (reduced PfPrx6^WT^), 32 430 m ^‒1^cm^‒1^ (reduced PfPrx6^H39Y^), 22 460 m ^‒1^cm^‒1^ (reduced hPrxVI^WT^, hPrxVI^C47S^, or hPrxVI^C91S^), 23 950 m ^‒1^cm^‒1^ (reduced hPrxVI^H39Y^), 11 460 m ^‒1^cm^‒1^ (reduced PfTrx1), or 9970 m ^‒1^cm^‒1^ (reduced PfGrx^C88S^). PfPrx6^WT^, PfPrx6^H39Y^, hPrxVI^WT^, hPrxVI^C47S^, hPrxVI^H39Y^, or hPrxVI^C91S^ were oxidized by incubation of the reduced enzymes with equimolar H_2_O_2_ for 30 min on ice. Oxidized PfPrx6^WT^, hPrxVI^C47S^, or hPrxVI^C91S^ were incubated with equimolar Na_2_S for 15 min on ice to generate the hydropersulfide species.

### Stopped‐Flow Kinetic Measurements

Kinetic measurements were carried out in a thermostatted SX‐20 spectrofluorometer (Applied Photophysics) at 25 °C. The change of tryptophan fluorescence was measured as total emission at an excitation wavelength of 295 nm with a slit width of 2 mm. All reagents were freshly dissolved in ice‐cold assay buffer. For the reaction of oxidized PfPrx6^WT^, PfPrx6^H39Y^, hPrxVI^WT^, hPrxVI^C47S^, hPrxVI^H39Y^, or hPrxVI^C91S^ with Na_2_S or Li_2_S, 0.5 µm freshly H_2_O_2_‐oxidized enzyme in syringe 1 was mixed with different concentrations of excess Na_2_S or Li_2_S in syringe 2. Alternatively, HS^−^ was freshly generated in situ by the addition of 1 M HCl to a 10 mM stock solution of TAA.^[^ ^59^ ^]^ The acidified stock solution was incubated at 60 °C for 1 h, followed by a stepwise dilution in assay buffer to a TAA concentration of 2–200 µm in syringe 1 before mixing with 0.5 µm freshly oxidized PfPrx6^WT^ in syringe 2. Traces of at least three technical replicates were averaged and fitted by double (or where indicated triple) exponential regression using the Pro‐Data SX software (Applied Photophysics) to obtain *k*_obs_ values. The *k*_obs_ values of three biological replicates from independent expression and protein purification experiments were plotted against the Na_2_S, Li_2_S, or TAA concentration in SigmaPlot 13.0 to obtain rate constants for each phase from linear fits. Single‐turnover experiments were performed with either one equivalent of H_2_O_2_ in the first syringe and reduced PfPrx6^WT^ and variable concentrations of Na_2_S in the second syringe or one equivalent of reduced PfPrx6^WT^ in the first syringe and freshly mixed H_2_O_2_ and Na_2_S (with an incubation time well under one minute) in the second syringe. The influence of the Trx or Grx/GSH systems was monitored by mixing 2 µm freshly H_2_O_2_‐oxidized PfPrx6^WT^ in syringe 1 with either 2 µm Na_2_S and up to 50 µm PfTrx1 or 2 µm Na_2_S, 10 mm GSH, and up to 50 µm PfGrx^C88S^ in syringe 2. Reactions of PfPrx6^WT^(SSH) were investigated by mixing 2 µm freshly Na_2_S‐incubated PfPrx6^WT^(SOH) in syringe 1 with up to 50 µm PfTrx1, up to 50 µm PfGrx^C88S^ with 10 mm GSH, up to 2 mm DTT, or up to 2 mm H_2_O_2_ in syringe 2.

### Mass Spectrometry

Freshly purified reduced, oxidized, or hydropersulfide hPrxVI^C91S^ or negative control hPrxVI^C47S^ were diluted in assay buffer to a final concentration of 1 µm and incubated at room temperature with either 5 mm Dim for 1 h or 20 µm MBB for 15 min. Whole protein electrospray mass spectrometry was conducted in positive ion mode using a liquid chromatography system with a POROS 10R1 column and a Bruker maXis time‐of‐flight spectrometer as described previously.^[^ ^34^ ^]^

### Statistical Analysis

For each stopped‐flow measurement, direct fluorescence traces of at least three technical replicates were averaged and fitted using the Pro‐Data SX software (Applied Photophysics) to obtain *k*_obs_ values. The *k*_obs_ values were plotted against the varied substrate concentration in SigmaPlot 13.0. Rate constants were determined from the slope or y‐axis intercept of linear fits in SigmaPlot 13.0. All data points were included. Mean values with standard deviation (± S.D.) of the *k*_obs_ values and of the rate constants from two or three biological replicates with at least three technical triplicates (*n* ≥ 2×3 or *n* ≥ 3×3) were calculated in Excel 2016.

## Conflict of Interest

The authors declare no conflict of interest.

## Author Contributions

L.La. and L.Le. contributed equally to this work as co‐first authors. L.La., L.Le., and L.B. performed the experiments and analyzed the data. M.D. and L.La. conceptualized the study. M.D. supervised the study. M.D. and L.La. wrote the manuscript. All authors read and approved the final version of the manuscript.

## Supporting information



Supporting Information

## Data Availability

The data that support the findings of this study are available from the corresponding author upon reasonable request.
