# Supplementary material for: H2S Is a Potential Universal Reducing Agent for Prx6‐Type Peroxiredoxins
Source: Adv Sci (Weinh). 2025 Oct 1;12(46):e07214. doi: 10.1002/advs.202507214 (PMC12697902; doi:10.1002/advs.202507214)
Supplement: Supplementary file 1 — Supporting Information [file ADVS-12-e07214-s001.docx]

Supporting Information

H_2_S is a potential universal reducing agent for Prx6-type peroxiredoxins

Lukas Lang,^1^ Laura Leiskau,^1^ Lea Bambach, and Marcel Deponte*

^1^ Lukas Lang and Laura Leiskau contributed equally to this work.

**Figure S1**

**
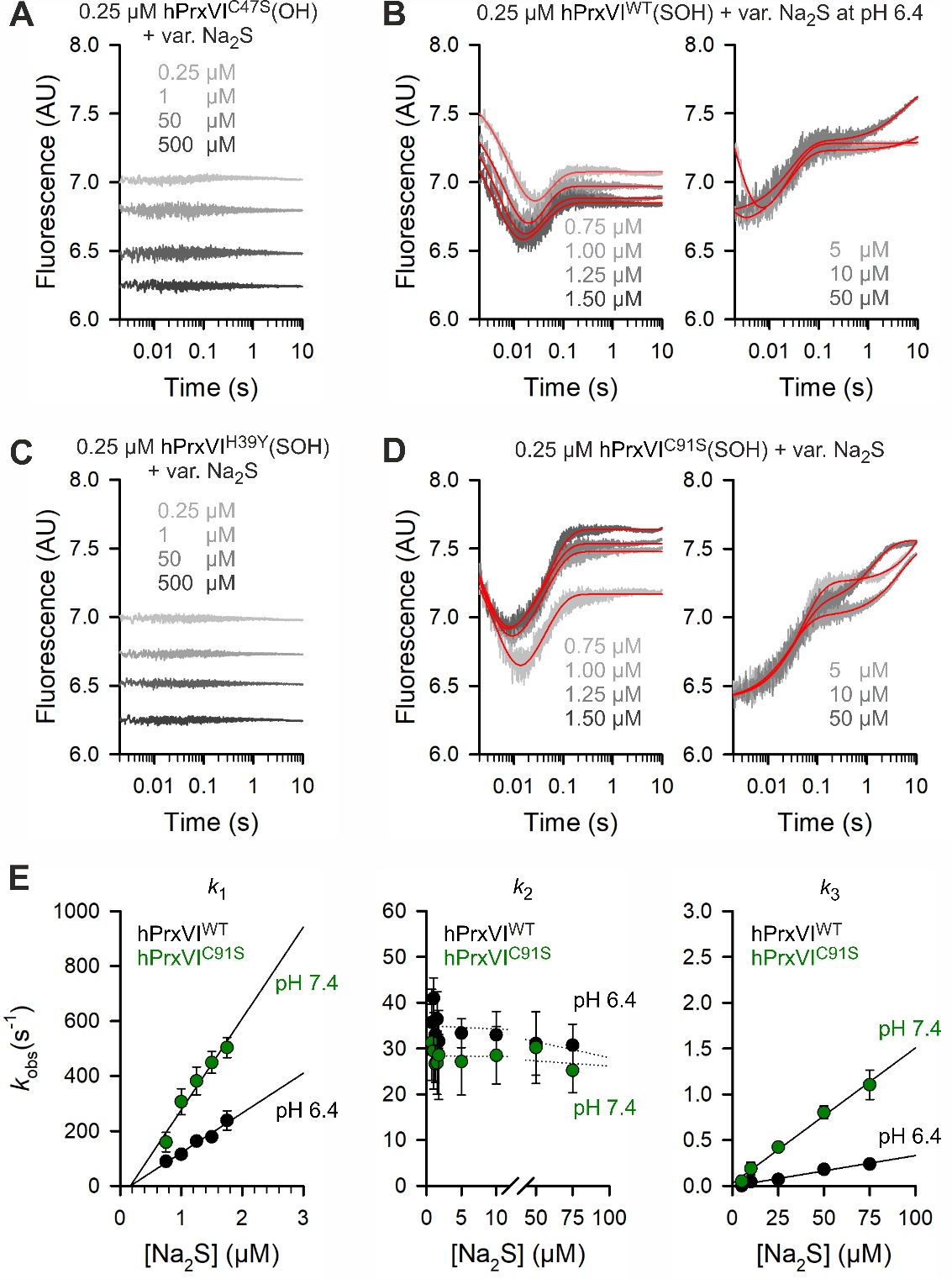
**

**Figure S1.** Mutant and pH control experiments for hPrxVI. A) Representative kinetic traces of stopped-flow measurements with oxidized active-site mutant hPrxVI^C47S^ and Na_2_S at pH 7.4 and 25°C. The result was confirmed in a biological replicate measurement (sample size n = 2×3). B) Representative kinetic traces of stopped-flow measurements with oxidized hPrxVI^WT^ at low (left panel) and high (right panel) Na_2_S concentrations at 25°C and a lower pH of 6.4. Double exponential fits are shown in red except for a triple exponential fit for the trace at 5 µM Na_2_S. C) Representative kinetic traces of stopped-flow measurements with oxidized hPrxVI^H39Y^ and Na_2_S at pH 7.4 and 25°C. The result was confirmed in a biological replicate measurement (sample size n = 2×3). D) Representative kinetic traces of stopped-flow measurements with oxidized hPrxVI^C91S^ at low (left panel) and high (right panel) concentrations of Na_2_S at pH 7.4 and 25°C. Double exponential fits are shown in red. All kinetic traces in panels A‒D were averaged from technical triplicate measurements. E) Secondary plots of the *k*_obs_ values with S.D. from the fits in panels B and D determined from three independent biological replicates including at least three technical replicates each (sample size n ≥ 3×3). Mean rate constants with S.D. were determined from the slopes or the y-axis intercepts of the linear fits from three independent biological replicates and are shown in Table 1.

**Figure S2**


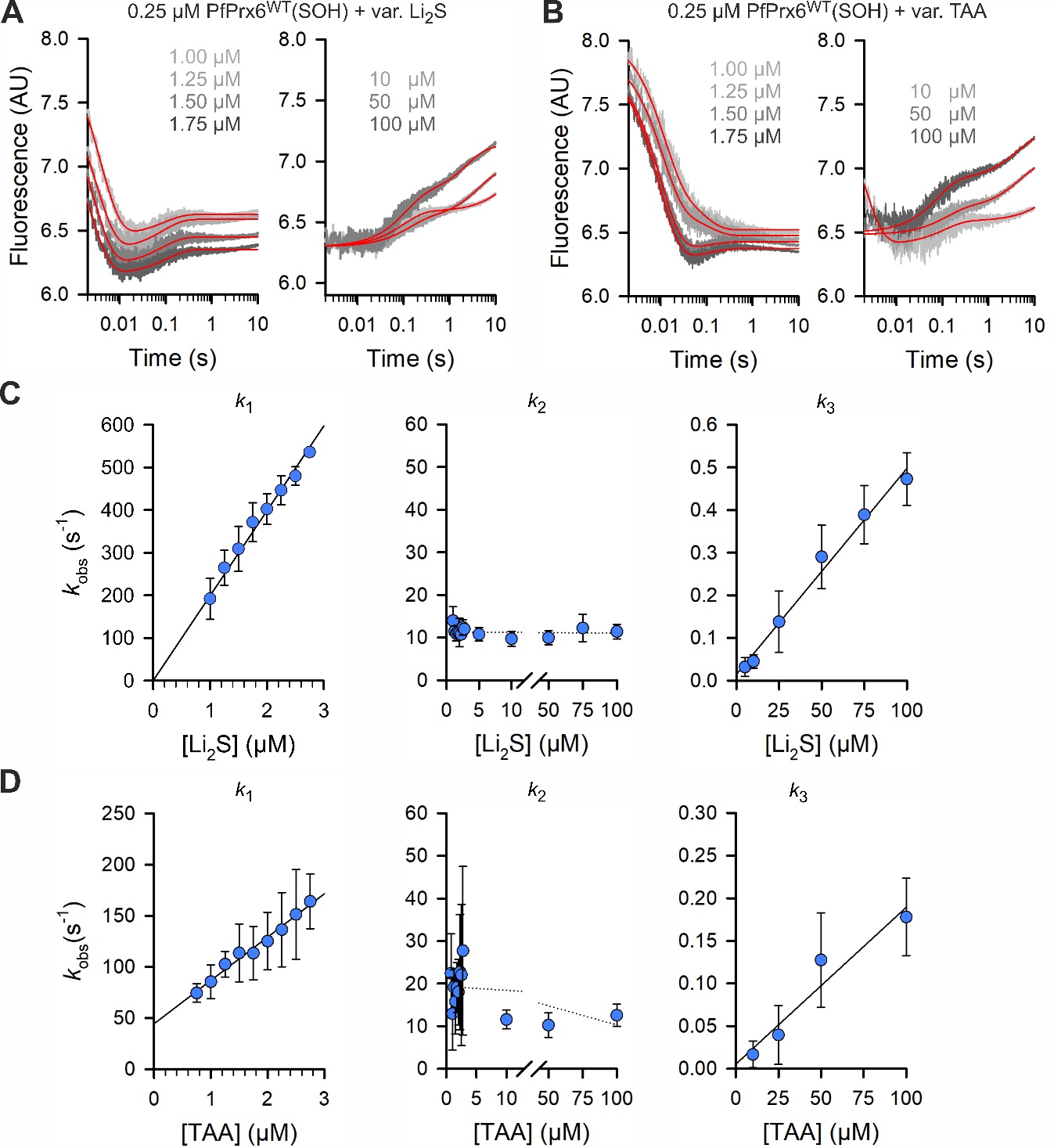


**Figure S2.** Rapid reduction of oxidized PfPrx6^WT^ by alternative HS^−^ sources. A) Representative kinetic traces of stopped-flow measurements with oxidized PfPrx6^WT^ at low (left panel) and high (right panel) concentrations of Li_2_S at pH 7.4 and 25°C. Double exponential fits are shown in red. B) Representative kinetic traces of stopped-flow measurements with oxidized PfPrx6^WT^ at low (left panel) and high (right panel) concentrations of acid-treated thioacetamide (TAA) at pH 7.4 and 25°C. Double exponential fits are shown in red except for a triple exponential fit for the trace at 10 µM TAA. All kinetic traces in panels A and B were averaged from technical triplicate measurements. C) and D) Secondary plots of the *k*_obs_ values with S.D. from the fits in panels A and B determined from three independent biological replicates including at least three technical replicates each (sample size n ≥ 3×3). Mean rate constants with S.D. were determined from the slopes or the y-axis intercepts of the linear fits from three independent biological replicates and are shown in Table 1.

**Figure S3**

**
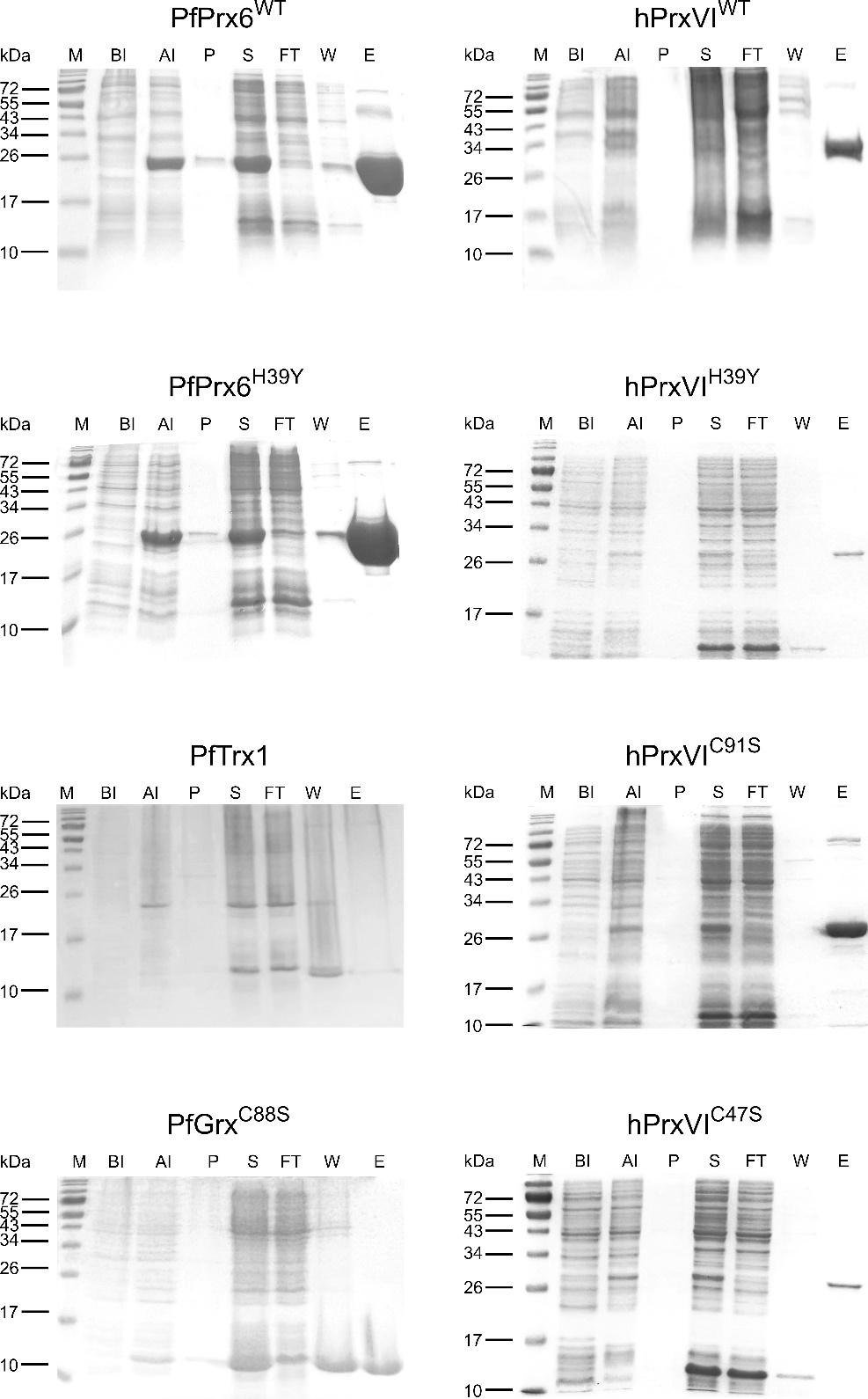
**

**Figure S3.** SDS-PAGE analysis of purified recombinant proteins. Shown are Coomassie-stained gels for representative purifications by Ni-NTA affinity chromatography for each of the indicated proteins. Fractions: M, marker; BI, before induction; AI, after induction; P, pellet after cell lysis; S, supernatant; FT, flow-through; W, wash; E, eluate. The calculated molecular masses are 26.2 kDa (PfPrx6^WT^ and PfPrx6^H39Y^), 13.0 kDa (PfTrx1), 13.7 kDa (PfGrx^C88S^), 26.2 kDa (hPrxVI ^WT^ and hPrxVI^H39Y^), and 26.1 kDa (hPrxVI^C91S^ and hPrxVI^C47S^).
